# Health impact of acute intermittent porphyria in latent and non-recurrent attacks patients

**DOI:** 10.1186/s13023-021-01742-3

**Published:** 2021-02-27

**Authors:** Juan Buendía-Martínez, María Barreda-Sánchez, Lidya Rodríguez-Peña, María Juliana Ballesta-Martínez, Vanesa López-González, María José Sánchez-Soler, Ana Teresa Serrano-Antón, María Elena Pérez-Tomás, Remedios Gil-Ferrer, Francisco Avilés-Plaza, Guillermo Glover-López, Carmen Carazo-Díaz, Encarna Guillén-Navarro

**Affiliations:** 1 Servicio de Neurología, Hospital General Universitario Morales Meseguer, Av Marqués de los Vélez, s/n, 30008 Murcia, Spain; 2 Instituto Murciano de Investigación Biosanitaria Virgen de la Arrixaca (IMIB-Arrixaca), Murcia, Spain; 3 Facultad de Ciencias de la Salud, Universidad Católica de Murcia (UCAM), Murcia, Spain; 4 Sección Genética Médica, Servicio de Pediatría, Hospital Clínico Universitario Virgen de la Arrixaca (HCUVA), Ctra. Madrid-Cartagena s/n., CP 30120 El Palmar (Murcia), Spain; 5 CIBERER-ISCIII, Madrid, Spain; 6 Servicio de Análisis Clínicos, HCUVA, Murcia, Spain; 7 Centro de Bioquímica y Genética Clínica, HCUVA, Murcia, Spain; 8 Applied Statistical Methods in Medical Research Group, UCAM, Murcia, Spain; 9 Departamento de Cirugía, Pediatría y Obstetricia y Ginecología, Facultad de Medicina, Universidad de Murcia (UMU), Murcia, Spain

**Keywords:** Acute intermittent porphyria, Chronic symptoms, Quality of life, Sporadic attacks, Chronic kidney disease

## Abstract

**Background:**

Acute intermittent porphyria (AIP) is a genetic disease characterized by acute neurovisceral attacks. Long-term clinical conditions, chronic symptoms and impaired health related quality of life (HRQoL) have been reported during non-attack periods but mainly in patients with recurrent attacks. Our aim was to investigate these aspects in sporadic AIP (SA-AIP) and latent AIP (L-AIP) patients. Fifty-five participants, 27 SA-AIP (< 4 attacks/year) and 28 L-AIP patients with a prevalent founder mutation from Spain were included. Medical records were reviewed, and individual interviews, physical examinations, biochemical analyses, and abdominal ultrasound scans were conducted. HRQoL was assessed through an EQ-5D-5L questionnaire. A comparative study was made between SA-AIP and L-AIP patients.

**Results:**

The earliest long-term clinical condition associated with SA-AIP was chronic kidney disease. Chronic symptoms were reported in 85.2 % of SA-AIP and 46.4 % of L-AIP patients. Unspecific abdominal pain, fatigue, muscle pain and insomnia were significantly more frequent in SA-AIP than in L-AIP patients. The EQ-5D-5L index was lower in SA-AIP (0.809 vs. 0.926, p = 0.0497), and the impact of “pain”, “anxiety-depression” and “mobility” was more intense in the EQ-5D-5L domains in SA-AIP than in L-AIP subjects and the general Spanish population.

**Conclusions:**

AIP remains a chronically symptomatic disease that adversely affects health and quality of life, even in patients with low rate of acute attacks. We suggest a regular monitoring of patients with symptomatic AIP regardless of their attack rate or the time since their last attack, with proper pain management and careful attention to kidney function.

## Background

Acute intermittent porphyria (AIP, MIM#176000) is the most common type of acute hepatic porphyrias (AHP) [1]. AIP is an autosomal dominant condition caused by a deficiency of hydroxymethylbilane synthase (HMBS; EC 2.5.1.61). Although most *HMBS* pathogenic variants are private, founder effects have been reported [2]. Such is the case with the *HMBS* pathogenic variant NM_000190.3(*HMBS*):c.669_698del30 p.(Glu223_Leu232) in the Region of Murcia, south-east Spain [3]. As a consequence, AIP prevalence has increased in this part of the country [4].

The clinical course of AIP is characterized by acute neurovisceral attacks involving the autonomic, peripheral and central nervous systems, with intervening non-attack periods [5, 6]. During acute attacks, the level of porphyrin precursors, aminolevulinic acid (ALA) and porphobilinogen (PBG) are markedly increased. Elevated urine PBG (U-PBG) is the main biochemical diagnostic marker of AHP attack. After recovery from a neurovisceral attack, the levels of urinary ALA (U-ALA) and U-PBG may remain high for long periods of time [7–9].

Most individuals with HMBS deficiency are latent AIP (L-AIP) subjects as they never experience neurovisceral attacks. However, manifest AIP patients may suffer from sporadic (< 4 per year) or recurrent (> 4 per year) neurovisceral attacks [10].

The most frequent long-term AIP-related clinical conditions are hypertension (HT), chronic kidney disease (CKD), and hepatocellular carcinoma (HCC) [11–13]. Chronic symptoms, such as abdominal and muscle pain, weakness, nausea, tiredness, anxiety and insomnia, have been described in the periods between acute attacks [14–19]. In addition, a decrease in health-related quality of life (HRQoL) has been reported in AHP patients compared to the general population, even in periods between attacks [14–20]. However, studies on these issues are scarce and those that exist mainly target patients with recurrent attack AHP (RA-AHP). Therefore, the aim of this study was to assess AIP impact on the health status during non-attack periods in sporadic attack patients (SA-AIP), which is the most common form of symptomatic AIP, and L-AIP subjects, focusing on chronic symptoms associated to the disease, HRQoL and AIP long-term clinical conditions.

## Materials and methods

### Patients

L-AIP subjects and SA-AIP patients from the Region of Murcia were included in this study after giving their written informed consent. All of them had a confirmed molecular AIP diagnosis, and were routinely followed in the Medical Genetics Section of the Hospital Clínico Universitario Virgen de la Arrixaca (HCUVA). This study was approved by the Ethical Committee of the HCUVA and all procedures were performed according to the Helsinki Declaration.

SA-AIP patients were defined by having had at least one previous neurovisceral attack and an annual rate of attacks lower than 4 per year. A neurovisceral attack was considered to have been suffered when the subject was admitted to hospital due to clinical compatible symptoms, with raised U-PBG levels, and received specific treatment. L-AIP status was defined when there was no neurovisceral attack background. Subjects were catalogued as hypertensive if they were under current antihypertensive treatment.

### Biochemical assessments

Spot U-ALA and U-PBG were assessed by spectrophotometry after column chromatography (BioSystems S.A., Barcelona, Spain), and results were normalized to urinary creatinine (U-ALA/Cr and U-PBG/Cr).

Serum liver enzymes (AST, ALT and GGT), serum ferritin and blood haemoglobin were determined by validated assays in the HCUVA clinical laboratory.

### Long-term clinical conditions and chronic symptoms

Clinical information was obtained by individual interview and a review of medical records. A self-made clinical questionnaire was used to identify chronic symptoms of interest. A physical examination and an abdominal ultrasound scan were conducted. CKD was considered with an estimated glomerular filtration rate (eGFR) lower than 60 mL/min per 1.73 m^2^, or with kidney damage markers, or both, in a minimum period of 3 months [21]. The eGFR was calculated using the CKD-EPI formula.

### HRQoL assesment

The EuroQoL 5-dimensions questionnaire 5-levels (EQ-5D-5L) was used to assess HRQoL [22]. The EQ-5D-5L measures five dimensions: “mobility”, “self-care”, “usual activities”, “pain/discomfort” and “anxiety/depression”. The 5 dimension scores are converted into a single index score ranging from 0 to 1 (EQ-5D-5L index) with “1” meaning optimal health. EQ-5D-5L includes a self-rated health assessment using a visual analogue score (VAS), with “100” signifying maximum and best health. Normative data from EQ-5D-5L in the Spanish population was available for comparison [23].

### Statistical approach

Continuous variables were summarized with means and standard deviations (SD), while qualitative variables were summarized with proportions. Multiple linear regression models were used to test associations between continuous variables and explanatory variables. Fisher’s tests were used to test associations between qualitative variables. Multiple logistic regression models were used to study association between U-ALA/Cr and U-PBG/Cr (exposures) and the different outcomes were coded as binary categories. All these models were adjusted by sex and age. Data were analysed using R (3.4.1. version) software package.

## Results

Of the 55 individuals included with confirmed *HMBS* pathogenic variants, 80 % carried the founder mutation NM_000190.3: c.669_698del30 p.(Glu223_Leu232). Other pathogenic variants were NM_000190.3:c.76 C > T p.Arg26Cys (8 patients, 14.54 %), NM_000190.3:c.750delA p.(Glu250GlufsTer4) (2 patients, 3.64 %) and NM_000190.3:c.275T > C p.(Leu92Pro) (1 patient, 1.82 %). Thirty-two (58.2 %) were females. Mean age was 44.7 years (16–77): 45.7 years (21–77) in women, and 43.9 years (16–75) in men. Results in SA-AIP and L-AIP patients are detailed in Table 1.

**Table 1:** Demographic, biochemical characteristics and HRQoL scores of SA-AIP and L-AIP patients

	SA-AIP	L-AIP	p value
N (%)	27 (49.1 %)	28 (50.9 %)	–
Age (mean ± SD)	47.5 ± 15.6	41.3 ± 15.7	–
Age range (years)	[28–77]	[16–75]	–
Females, N (%)	20 (74.1 %)	12 (42.8 %)	–
Males, N (%)	7 (25.9 %)	18 (57.1 %)	–
c.669_698del30 carriers^a^, N (%)	20 (74.1 %)	24 (85.7 %)	–
BMI (mean ± SD)	26.93 ± 4.4	28.17 ± 4.93	0.3436
*Biochemical assessments* (mean ± SD)			
eGFR (ml/min per 1.73m^2^)	73.16 ± 27.3	105.07 ± 19.2	< 0.0001*
AST (U/L)	28.19 ± 6.5	22.31 ± 8.6	0.0082*
ALT (U/L)	27.04 ± 14.4	25.3 ± 10.8	0.6119
GGT (U/L)	20.24 ± 18.5	26.52 ± 17.6	0.3047
Hb in males (g/dl)	13.54 ± 1.73	14.4 ± 1.36	0.0563
Ferritin in males (ng/ml)	57.59 ± 64.26	80.46 ± 65.08	0.2373
Hb in females (g/dl)	12.64 ± 0.89	13.34 ± 0.95	0.1270
Ferritin in females (ng/ml)	53.44 ± 37.5	43.73 ± 68.7	0.6513
U-PBG/Cr^b^ (µmol /mmol)	17.09 ± 15.93	1.32 ± 2.29	0.0001*
U-ALA/Cr^b^ (µmol /mmol)	9.86 ± 6.76	6.06 ± 4.35	0.1333
*HRQoL scores* (mean ± SD)			
EQ-5D-5L index	0.809 ± 0.16	0.926 ± 0.14	0.0497*
EQ-5D-5L VAS	75.44 ± 16.16	86.78 ± 15.78	0.1353

*SA-AIP* sporadic attack AIP, *L-AIP* latent AIP, *N* total number of participants, *BMI* body mass index, *eGFR* estimated glomerular filtrate rate, *AST* amino aspartate transferase, *ALT* alanine aminotransferase, *GGT* gamma glutamyl transferase, *Hb* haemoglobin; ferritin; *U-PBG/Cr* urinary porphobilinogen/creatinine ratio, *U-ALA/Cr* urinary aminolevulinic acid/creatinine ratio, *VAS* visual analogue scale

*P value ≤ 0.05 adjusted by age and gender. Laboratory reference ranges: eGFR > 60mL/min/1.73m2; AST 5–35 U/L; ALT 10–40 U/L; GGT 7–50 U/L; Hb 12–16 g/dL; Ferritin 12–300 ng/mL; U-PBG/Cr < 1.5 µmol /mmol; U-ALA/Cr < 3.8 µmol /mmol

^a^Founder mutation in the Region of Murcia. ^b^Only evaluated in 51 participants

Twenty-seven patients (49.1 %) had a history of SA-AIP and 28 (50.9 %) were L-AIP, with mean ages of 47.5 years (28–77) and 41.3 years (16–75) respectively. There was higher proportion of females in the SA-AIP group (74.1 %, 20/27) than in L-AIP (42.8 %, 12/28). The mean background number of neurovisceral attacks per patient was 2.27 (SD 1.51): 2.45 (1–6, SD 1.5) in women, and 1.66 (1–3, SD 0.98) in men. Most SA-AIP patients (81.5 %, 22/27) had suffered only 3 or less attacks in their lifetime. 85.2 % (23/27) had had no attacks in the year preceding this study (average number of attacks in this period was 0.15 per patient). No patient had suffered an attack in the last 6 months and the average time since the last attack was 16.17 years (SD 12.6).

There were no statistical differences in the number of background neurovisceral attacks between founder mutation carriers and carriers of the rest of the pathogenic variants.

### Long-term clinical conditions

**Fig. 1:**
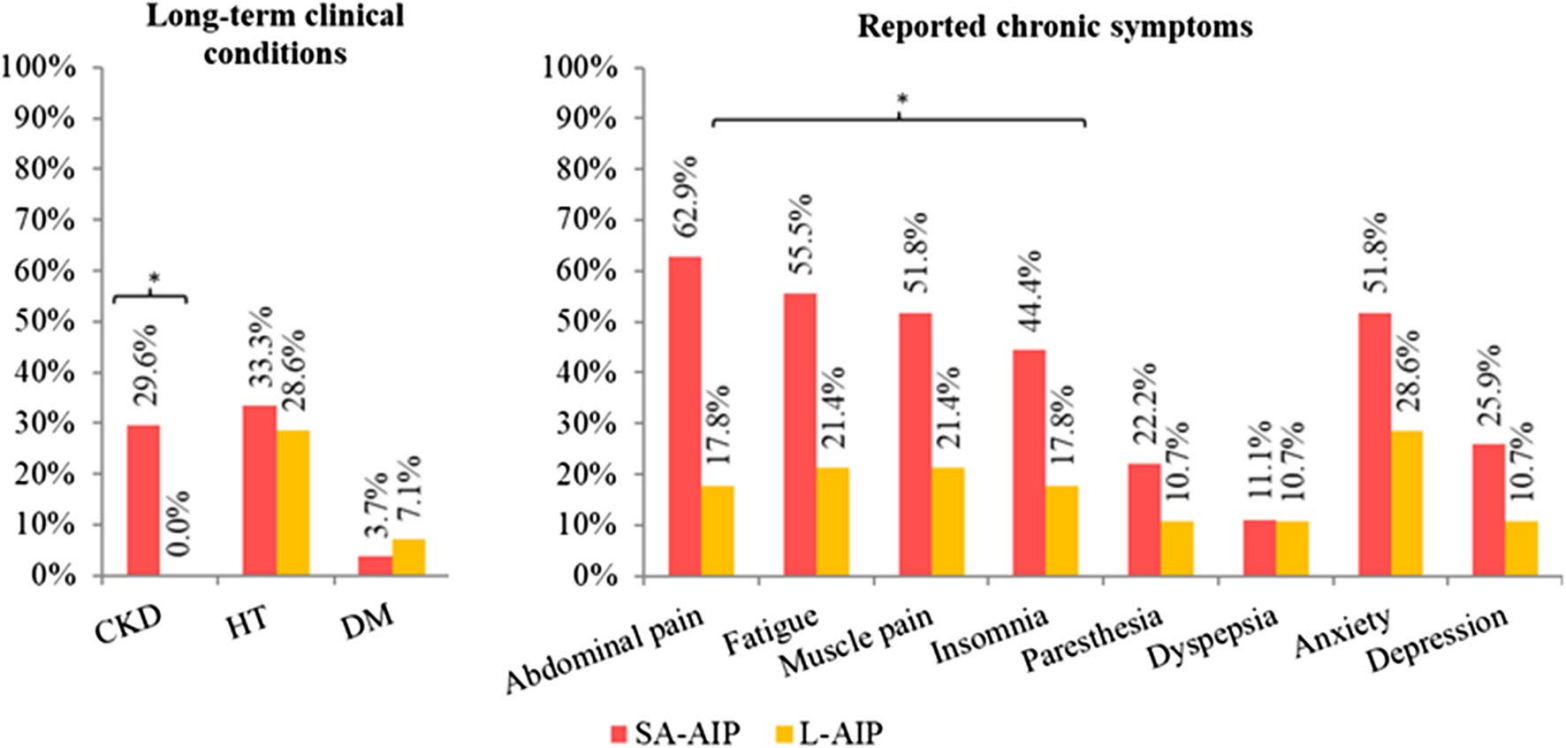
Long-term clinical conditions and reported chronic symptoms in SA-AIP and L-AIP patients. Asterisk indicates significant differences when comparing SA-AIP and L-AIP (p value ≤ 0.05 adjusted by age and gender, the respective values are shown in the text). *SA-AIP* sporadic attack AIP, *L-AIP* latent AIP

Main clinical conditions in SA-AIP and L-AIP are shown in Fig. 1. 14.5 % (8/55) of AIP individuals had received a previous CKD diagnosis at the mean age of 34.6 years (19–55). All of them had SA-AIP (8/27), showing significant differences in the frequency of CKD compared to L-AIP (p = 0018). Accordingly, SA-AIP patients had a significantly lower eGFR than L-AIP subjects (p < 0.0001) (Table 1). Proteinuria, if present, was below 200 mg/g creatinine (0–192) in all cases.

30.9 % of subjects (17/55) were currently following antihypertensive treatment. There was no substantial difference in the prevalence of HT in SA-AIP and L-AIP (Fig. 1), nor in the age of onset (SA-AIP: 47.44 ± 17.78 years; L-AIP: 47.25 ± 5.82 years; p = 0.9759) or the number of antihypertensive agents needed for the proper control (SA-AIP: 1.44 ± 0.73; L-AIP: 1.88 ± 1.13; p = 0.3741).

Serum liver enzymes were above normal levels in 11,1 % (3/27) of SA-AIP and 3 % (1/27) of L-AIP subjects, although the average levels of ALT, AST and GGT were within normal limits in both sets of patients (Table 1). AST was significantly higher in SA-AIP patients than in L-AIP. No hepatic or other abdominal abnormalities were detected by ultrasound examination. Serum ferritin and haemoglobin levels were similar in the SA-AIP and L-AIP groups.

### Reported chronic symptoms

Chronic symptoms were reported in 85.2 % (23/27) of SA-AIP patients and 46.4 % (13/28) of L-AIP. Recurrent non-specific abdominal pain followed by fatigue, muscle pain, anxiety and insomnia, were the most prevalent symptoms in SA-AIP with a significantly higher frequency than in L-AIP (p = 0.0023, p = 0.0129, p = 0.0261, p = 0.1022, p = 0.0437, respectively), with the exception of anxiety which, followed by fatigue and muscle pain, were the most frequent symptoms in L-AIP (Fig. 1). Anxiety was the main psychiatric problem in both groups, while depressive symptoms less frequent.

### HRQoL assessment

**Fig. 2:**
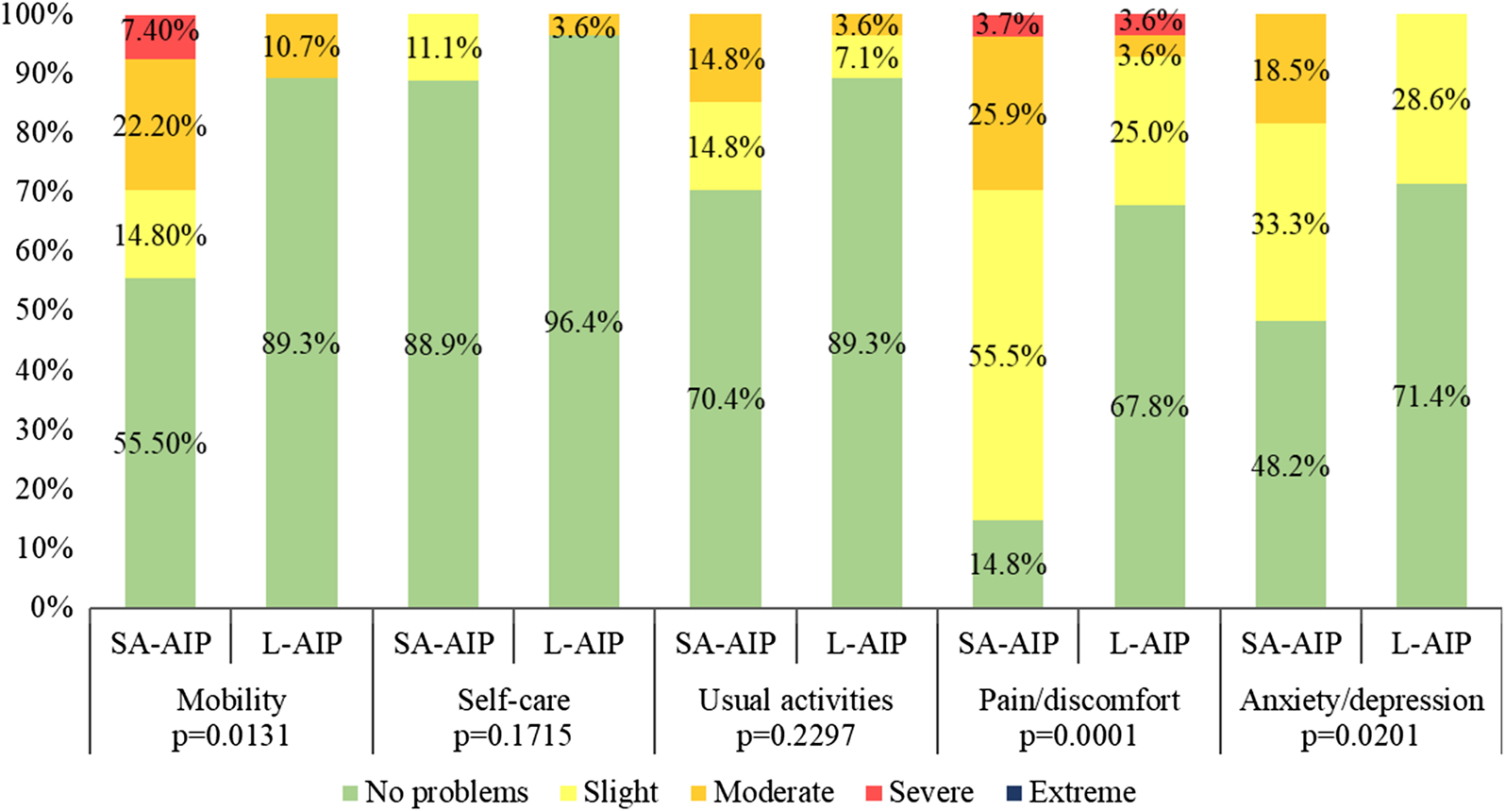
EQ-5D-5L domains scores in SA-AIP and L-AIP patients. Fisher's tests revealed greater severity in mobility, pain/discomfort and anxiety/depression problems in SA-AIP patients than in L-AIP individuals. *SA-AIP* sporadic attack AIP, *L-AIP* latent AIP

EQ-5D-5L data analysis showed that “mobility”, “pain/discomfort” and “anxiety/depression” domains had significantly worse intensity in SA-AIP than L-AIP (Fig. 2).

Pain perception was greater in SA-AIP than in L-AIP (p = 0.0001). Out of 27 SA-AIP patients, 23 (85.2 %) suffered pain to some degree, and 8 (29.6 %) to a moderate or severe degree (Fig. 2). In contrast, 32.2 % (9/28) of L-AIP subjects reported pain, but only 7.2 % to a “more than slight” degree. Anxiety/depression problems were also of greater intensity among SA-AIP patients (p = 0.0201) since L-AIP subjects had only slight symptoms. Meanwhile, 18.5 % (5/27) of SA-AIP patients reported moderate anxiety/depression problems. Finally, “mobility problems” were of greater severity in SA-AIP than in L-AIP (p = 0.0131), with 44.4 % (12/27) of SA-AIP patients reporting mobility problems, 29.6 % (8/27) to a moderate or severe degree. Analysis of the causes of impaired mobility showed that arthritis, previous stroke sequel or chemotherapy-induced peripheral neuropathy were some of the causes, but the main cause in SA-AIP patients with moderate or severe grade problems was a residual axonal polyneuropathy associated with a previous AIP acute attack (5/8, 62.5 %).

The mean EQ-5D-5L index and VAS were on average worse in SA-AIP than in L-AIP (Table 1). When EQ-5D-5L index was compared by percentiles with those in Spanish normative population by age ranges (Table 2), lower indexes in SA-AIP and minor differences with L-AIP were observed. The lowest EQ-5D-5L index in SA-AIP outstand, especially in the 25–34 and the 35–44 age ranges, which are below the 10th percentile.

**Table 2:** Spanish normative population, SA-AIP and L-AIP EQ-5D-5L index values expressed by age ranges

Age range	Spanish normative values^a^	SA-AIP			L-AIP		
	EQ-5D-5L index	EQ-5D-5L index	N	Percentile^b^	EQ-5D-5L index	N	Percentile^b^
18–24	0.983 (0.07)	–	–	–	1.00 (0.00)	5	>P95
25–34	0.977 (0.07)	0.880 (0.10)	4	P5–P10	0.970 (0.05)	6	P50–P60
35–44	0.957 (0.11)	0.808 (0.13)	11	P5–P10	0.943 (0.08)	5	P20–P30
45–54	0.934 (0.14)	0.940 (0.05)	3	P30–P40	0.971 (0.06)	5	P50–P60
55–64	0.904 (0.17)	0.743 (0.15)	6	P10–P20	0.796 (0.25)	4	P10–P20
65–74	0.870 (0.20)	0.846 (0.09)	2	P10–P20	0.811 (0.22)	3	P20–P30
75–84	0.785 (0.24)	0.342	1	P5–P10	–	–	–

Values are reported as mean (SD)

*N* total number of individuals, *SA-AIP* sporadic attack AIP, *L-AIP* latent AIP

^a^Spanish normative values for EQ-5D-5L index and percentiles were extracted from Hernandez, 2018 [23], ^b^Percentile of the EQ-5D-5L index with respect to the Spanish normative values

**Fig. 3:**
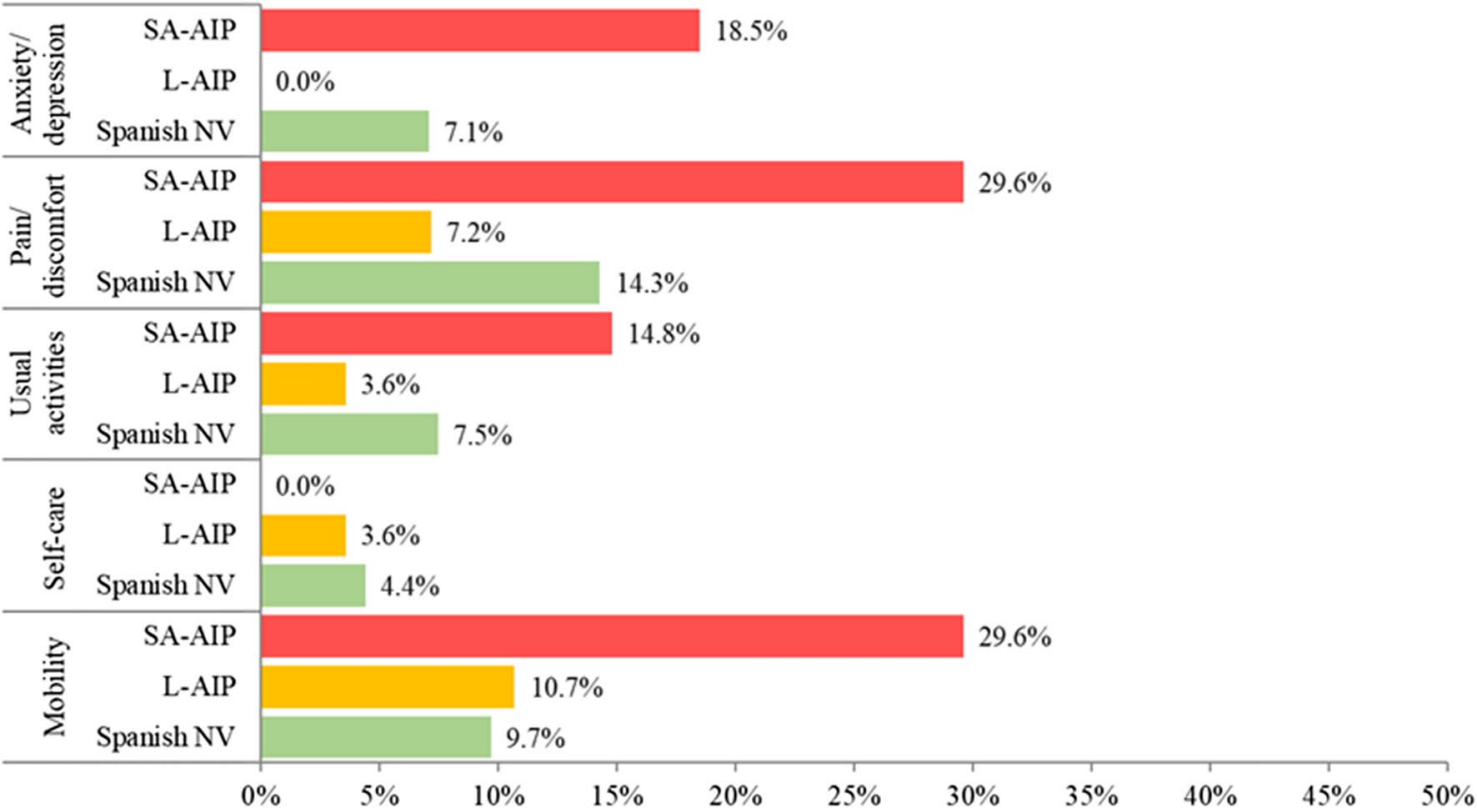
Categorized score (including: moderate, severe or extreme scores) in EQ-5D-5L domains. *Spanish NV* Spanish normative population, *L-AIP* latent AIP, *SA-AIP* sporadic attack AIP

With regard to the domains included in the EQ-5D-5L questionnaire, Fig. 3 shows the proportion of patients who reported more than “slight problems” for each level, meaning that only levels “moderate problems,“ “severe problems,“ and “extreme/unable” were considered. The published results available in the Spanish normative population have also been included. There was a higher proportion of SA-AIP patients with “mobility”, “pain/discomfort”, “anxiety/depression” and “usual activities” problems than L-AIP patients or individuals in the normative population. The latter two groups had similar proportions in the different domains, except for a slightly higher proportion of problems in the “pain/discomfort” and “usual activities” domains, observed in L-AIP.

### Urine ALA and PBG assessments

Urine samples were collected from 51 individuals (24 SA-AIP and 27 L-AIP). The U-PBG/Cr, but not U-ALA/Cr, was significantly higher in SA-AIP than in L-AIP (Table 1). In SA-AIP patients, U-PBG/Cr values correlate with U-ALA/Cr levels: for each unit increase in U-ALA/Cr, there was an average increase of 2.06 U-PBG/Cr units (CI 95 % 1.7–2.4, p < 0.001). In L-AIP no significant association was found between U-ALA /Cr and U-PBG/Cr; and higher U-ALA/Cr levels did not correlate with higher levels of U-PBG/Cr, whose values remained similar among these patients (data not shown).

A regression study was carried out to assess a possible relationship between U-ALA/Cr and U-PBG/Cr, on the one hand, and fatigue, muscle or abdominal pain and the level of anxiety and pain on the other (EQ-5D-5L data, classified as “absent or slight problems” and “more than slight problems”). No significant relationship was found between the levels of porphyrin precursors and the aforementioned chronic symptoms.

## Discussion

To the best of our knowledge, this is the first study describing chronic manifestations in an AIP population with SA-AIP, which represents the majority of patients with symptomatic AIP, and L-AIP. The findings show that long-term clinical conditions and chronic symptoms more frequently occur in SA-AIP patients compared with L-AIP. Also, the quality of life of patients with SA-AIP was seen to be more impaired compared with L-AIP patients.

As described by Pallet et al. [12], impaired kidney function and CKD was strongly associated with SA-AIP condition. This suggests that there may be an increased risk of CKD even in patients who have only had one or two acute attacks in their lives, as was the case in this study. This relation is reinforced by the fact that the age of CKD diagnosis and the age of first AIP attack were quite close. In addition, the prevalence of renal impairment in SA-AIP resembles that seen in other AHP cohorts [19].

In contrast to other investigations [11, 12, 24, 25], HT prevalence was similar to that described in the Spanish reference population [26], and there was no difference between SA-AIP and L-AIP. Pallet et al. described HT as a frequent complication in AIP patients with symptoms [12], but the average age of their cohort was substantially higher (65 years) than ours.

To date, no cases of HCC have been diagnosed, and biochemical and ultrasound examination have not revealed any other liver abnormalities. It should be noted that the participants in this study had an average age of less than 50 years, the age above which an increased risk of HCC has been described [27, 28]. Longer-term follow-up and a larger cohort may be needed to appreciate the overall relationship of SA-AIP and L-AIP with HT, liver function and HCC risk.

The available published data had shown that HRQoL is impaired in AIP, and self-reported chronic symptoms range from 18 to 22 % in patients with less severe AHP to 65–78 % in patients with RA-AHP [15, 17–19]. Overall, we found that the frequency of chronic symptoms in SA-AIP patients (85.2 %) was almost twice than that of L-AIP patients (46.4 %).

The differences in pain symptom frequencies between SA-AIP and L-AIP are noteworthy. Pain, mainly recurrent and non-specific abdominal pain and/or generalized muscle pain, should be considered as one of the main chronic symptoms associated with symptomatic AIP, as it was in the EQ-5D-5L questionnaire. Fatigue (excluding iron deficiency anaemia as a possible cause of fatigue) and insomnia were also more relevant problems in SA-AIP than in L-AIP. Anxiety, which was also shown to be a common symptom, was not significantly more frequent in the SA-AIP than in the L-AIP, although it was more intense in the former on the basis of the results of the EQ-5D-5L questionnaire. In general, the symptoms in SA-AIP and their extent appear similar to those reported in RA-AHP [17–19].

EQ-5D-5L also revealed more mobility problems in SA-AIP patients than in L-AIP, which may have been due to AIP axonal polyneuropathy sequels in our SA-AIP group. AIP related acute neuropathy was no progressive and the patients who suffered from it tended to improve with slight sequels.

All of the above is consistent with worse health status reported in SA-AIP than in L-AIP patients, through the 5Q-5D-5L index and VAS. This suggests, as expected, that overall health status is related with the frequency of acute attacks and disease activity. Although HRQoL studies that include a healthy control group would be needed, here we also show evidence of a worse QoL reported by SA-AIP patients than that published in the Spanish normative population.

Finally, higher U-PBG/Cr levels were found in SA-AIP patients than in L-AIP patients, as already known [29]. This may be explained by increased disease activity in SA-AIP and because porphyrins precursors remain elevated for long periods of time after an acute attack. As has been reported before, U-PBG/Cr levels in SA-AIP patients were proportional to those of U-ALA/Cr [7]. Since U-PBG/Cr was not related to U-ALA/Cr in L-AIP (even in subjects with high U-ALA/Cr), this finding may indicate that apart from HBMS deficiency (present in L-AIP and SA-AIP), there is a sustained dysfunction in the haem metabolic pathway of patients with SA-AIP. These data must be carefully interpreted and further research is needed to assess this hypothesis and its possible relationship with the development of chronic symptoms.

Since the average time without attacks in SA-AIP patients was more than 15 years, in our opinion our findings should not be attributed to possible sequels or manifestations associated with recent attacks, which could lead to a bias in the study. It is important to note that mean age in the SA-AIP sample was 47.5 years. This is still a relatively young population, and the full chronic impact of the disease may not have fully developed. Conversely, this means that the findings observed in this study may be considered as early-onset manifestations. This hypothesis is reinforced by the worse EQ-5D-5L index results in the young adult age ranges observed in SA-AIP compared to the normative population.

However, these findings should be corroborated by further studies in larger SA-AIP populations. In our opinion, it is worth extending the studies related with these aspects, which until now have been carried out mainly in patients with recurrent attacks, to patients who are subject to sporadic attacks.

## Conclusions

In summary, the present study shows that AIP has a negative impact on health, evidencing that patients with SA-AIP have significant chronic symptoms and long-term clinical conditions compared to L-AIP, despite low attack rates or long periods without attacks.

AIP should not be considered only as an acute disease, since neurovisceral attacks are not the only clinical expression of the disease.

This reinforces the need for regular monitoring of patients with symptomatic AIP regardless of their attack rate or the time since their last attack. Special attention should be paid to kidney function in order to detect early deterioration and progression to CKD.

## Data Availability

The datasets used and/or analysed during the current study are available from the corresponding author on reasonable request.

## Abbreviations

AHP: Acute hepatic porphyria
AIP: Acute intermittent porphyria
Cr: Creatinine
CKD: Chronic kidney disease
eGFR: Estimated glomerular filtration rate
EQ-5D-5L: EuroQoL 5-dimensions questionnaire 5-levels
HCC: Hepatocellular carcinoma
HMBS: Hydroxymethylbilane synthase
HRQoL: Health related quality of life
HT: Hypertension
L-AIP: Latent acute intermittent porphyria
RA-AHP: Recurrent attack acute hepatic porphyria
SA-AIP: Acute intermittent porphyria
U-ALA: Urine aminolevulinic acid
U-PBG: Urine porphobilinogen
VAS: Visual analogue score
